# Histone deacetylase inhibitory effect of Brazilian propolis and its association with the antitumor effect in Neuro2a cells

**DOI:** 10.1002/fsn3.131

**Published:** 2014-06-02

**Authors:** Shinobu Ishiai, Wataru Tahara, Etsuko Yamamoto, Rindai Yamamoto, Kaoru Nagai

**Affiliations:** 1 Interdisciplinary Graduate School of Medicine and Engineering, University of YamanashiChuo-shi, Yamanashi, Japan; 2 Nihon Natural Foods Co., Ltd.Tokyo, Japan

**Keywords:** Brazilian propolis, cell cycle arrest, cell death, Hdac, Neuro2a

## Abstract

Propolis is a resinous product produced by honey bees and is known to have antitumor functions. On the other hand, histone deacetylase (Hdac) inhibitors have recently attracted attention for their antitumor effects. In this study, we examined whether Brazilian green propolis has an Hdac inhibitory activity and its contribution on antitumor effects. By in vitro Hdac activity assay, Brazilian propolis extract (BPE) significantly inhibited the enzyme activity. Actually, BPE treatment increased the intracellular histone acetylation in Neuro2a cells. Regarding antitumor effect in Neuro2a cells, BPE treatment significantly decreased cell viability. An Hdac activator theophylline significantly attenuated the effect. Then, we analyzed whether the decreasing effect on cell number was caused by cell death or growth retardation. By live/dead cell staining, BPE treatment significantly increased the dead cell number. By cell cycle analysis, BPE treatment retarded cell cycle at the M-phase. Both of these cellular effects were suppressed by addition of theophylline. These data indicate that BPE induced both cell death and growth retardation via Hdac inhibitory activity. We demonstrated that Brazilian propolis bears regulatory functions on histone acetylation via Hdac inhibition, and the effect contributes antitumor functions. Our data suggest that intake of Brazilian propolis shows preventing effects against cancer.

Propolis is a resinous product produced by honey bees (*Apis mellifera*), and is used to seal cracks and holes in the hive. It is thought that propolis functions to protect the bee colony against bacteria, fungi, and viruses. Propolis is used as a folk medicine in many regions of the world. It has been reported that propolis contains many chemical compounds, such as flavonoids, phenolic acids and esters, substituted phenolic esters, terpenoids, *β*-steroids, and aromatic aldehydes and alcohols (Banskota et al. 2001). Propolis is known to show a broad range of biological activities, such as anti-inflammatory (Paulino et al. 2006), antioxidant (Nakanishi et al. 2003), antimicrobial (Salomão et al. 2008), and antitumor (Burdock 1998) effects. However, the chemical compositions and the functions depend on produced area and botanical sources. Brazilian green propolis, which botanical source is *Baccharis dracunculifolia* DC (Asteraceae), is used as a health food in Europe and Japan. *Baccharis dracunculifolia* has been reported to contain many biologically active compounds, such as artepillin C, baccharin, and caffeic acid (de Sousa et al. 2011). Thus, Brazilian green propolis is expected to contain these biologically active compounds. The antitumor property of Brazilian green propolis was reported in several studies (Kimoto et al. 1998; Li et al. 2007; Búfalo et al. 2009). It was reported that the propolis induced apoptotic cell death via TRAIL-dependent signaling (Sawicka et al. 2012).

Acetylation of histones is one of the crucial parts of the epigenetic transcriptional regulation. Histone acetyltransferase (Hat) and histone deacetylase (Hdac) control the balance of histone acetylation (Yang and Seto 2007). Acetylation at lysine residues neutralizes the positive charge and weakens the interaction between histone and DNA. That induces opened chromatin structure which is accessible to transcriptional factors. Hence, deacetylation by Hdac induces a closed chromatin structure which is a transcriptionally inactive state. In four classes, 18 of Hdacs have been identified in mammals (de Ruijter et al. 2003). Class I Hdacs have been reported to regulate many gene expressions (Dokmanovic et al. 2007). It means that inhibition of class I Hdacs affects many gene expressions. In cancer cells, the alterations of gene expressions by Hdac inhibitors have been reported to show an antitumor effect, such as cell cycle arrest and apoptosis (de Ruijter et al. 2003; Dokmanovic et al. 2007). Practically, the Food and Drug Administration accepted two Hdac inhibitors suberoylanilide hydroxamic acid (SAHA) and FK-228 for the treatment of cutaneous T-cell lymphoma, and several Hdac inhibitors are in phase I or II of clinical trials in cancer patients (Monneret 2005).

Recently, some natural products such as short-chain fatty acids and some polyphenols have been reported to inhibit Hdac activity (Link et al. 2010). Since propolis contains analogs of previously reported Hdac inhibitory molecules (Banskota et al. 2001), it is assumed that propolis inhibits Hdac activity. Taiwanese and Chinese propolis and its components have been reported to show Hdac inhibitory activity (Huang et al. 2012; Sun et al. 2012). However, since the chemical compositions of propolis are different between produced areas, there is no guarantee that Brazilian green propolis also shows an Hdac inhibitory activity. In this study, we analyzed whether Brazilian green propolis has an Hdac inhibitory activity and the inhibitory activity associates with the antitumor function.

First, we evaluated whether ethanolic extract of Brazilian propolis (BPE) inhibits class I Hdac enzyme activity in vitro. Hdac inhibitory activity was determined by an HDACs deacetylase fluorometric assay kit (CycLex, Nagano, Japan) under the manufacturer's instruction (for detailed methods, see Data S1). Amounts of 100, 200, and 500 *μ*g/mL of BPE significantly decreased the relative activities to 85.8 ± 5.8%, 64.8 ± 4.9%, and 24.8 ± 0.3% compared to the control, respectively (Fig. 1A). Our data indicate that BPE directly inhibits class I Hdac enzyme activity and the inhibitory activity at 500 *μ*g/mL is a similar level to commonly used pan-Hdac inhibitor 1 mmol/L sodium butyrate (SB) (Fig. 1A). Then, we examined whether BPE treatment actually affects intracellular histone acetylation in mouse neuroblastoma Neuro2a cells. The cells were treated with 100 or 200 *μ*g/mL of BPE for 6 h and the level of intracellular histone acetylation was quantitated by a cell-based ELISA kit (CycLex, Nagano, Japan) under the manufacturer's instruction (for detailed methods, see Data S1). Amount of 200 *μ*g/mL of BPE treatment significantly increased the relative acetylation to 170.5 ± 17.8% compared to the control (Fig. 1B). In addition, we analyzed the level of histone H3 acetylation in the treated cells by Western blot analysis (for detailed methods, see Data S1) and the acetyl H3/total H3 were quantitated by using C-DiGit-image studio (LI-COR, Lincoln, NE). Cells treated with 200 *μ*g/mL of BPE significantly increased acetylated histone H3 protein to 203.0 ± 24.6% compared to untreated cells (Fig. 1C and D). These data indicate that BPE actually acts as an Hdac inhibitor intracellularly. Hdac inhibitors are generally classified into short-chain fatty acids, benzamides, hydroxamic acids, or cyclic peptides (Wu et al. 2012). Brazilian green propolis has been reported to contain these analogs, a few of which have already been reported to show Hdac inhibitory activities, such as kaempferol, chrysin, and p-coumaric acid (Waldecker et al. 2008; Pal-Bhadra et al. 2012; Berger et al. 2013). However, these compounds are minor components in Brazilian green propolis. The contents of kaempferol, chrysin, and p-coumaric acid were ∼0.4, 1.8, and 8.5 mg/g in the BPE, respectively (Li et al. 2007). However, 50% Hdac inhibitory concentrations of these compounds are higher than 50 *μ*mol/L (Waldecker et al. 2008; Pal-Bhadra et al. 2012; Berger et al. 2013). It indicates that the contents of these compounds are not enough to explain the inhibitory activity of BPE, while artepillin C, one of the major components of BPE, did not show Hdac inhibitory activity in vitro (data not shown). It suggests that BPE contains some other unknown Hdac inhibitory molecules. From these data, we first found that Brazilian green propolis has an Hdac inhibitory activity which affects epigenetic transcriptional regulation and suggests the presence of novel Hdac inhibitory molecules.

**Figure 1:**
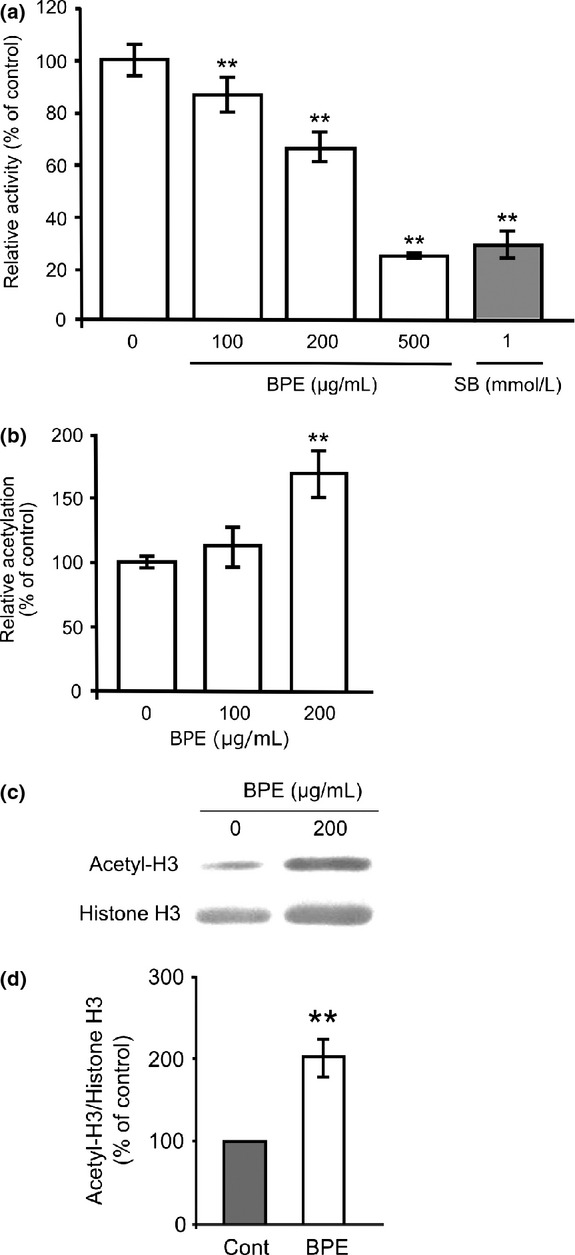
Effects of BPE on class I Hdac enzyme activity and cellular histone acetylation. (A) Hdac activity was measured in the presence of BPE or sodium butyrate (SB). The results are presented as mean ± SD of relative activity compared to the control (*n* = 6). **P < 0.01, ANOVA. (B) Neuro2a cells were treated with 100 or 200 *μ*g/mL of BPE for 6 h and subjected to quantitative analysis of histone acetylation. The results are presented as mean ± SD of relative activity compared to the control (*n* = 6). **P < 0.01, ANOVA. (C) Neuro2a cells were treated with 200 *μ*g/mL BPE for 6 h. Then, cell lysates were subjected to western blot analysis against acetylated (Acetyl-H3) or total (Histone H3) histone H3. (D) The expression levels of Acetyl-H3/Histone H3 were quantitated by densitometric analysis. The results are presented as mean ± SD of relative expression level compared to untreated cells (*n* = 3). **P < 0.01, ANOVA. BPE, Brazilian propolis extract; Hdac, histone deacetylase; ANOVA, analysis of variance.

Next, we evaluated whether Hdac inhibitory activity of BPE is involved in the antitumor function. Neuro2a cells were cultured at 30,000 cells/well in a 24-well plate. After 24 h of culture, the cells were treated with 200 *μ*g/mL of BPE in the presence or absence of 100 *μ*mol/L Hdac activator theophylline for 24 h and then live cells were counted by the trypan blue exclusion assay. A treatment of 200 *μ*g/mL of BPE significantly decreased the relative live cell number to 39.5 ± 3.0% compared to the control. In the presence of theophylline, the effect of BPE was significantly attenuated to 49.7 ± 6.6% (Fig. 2A). Moreover, we verified whether commonly used Hdac inhibitors SAHA and SB actually show the antitumor effect via the Hdac inhibitory activities. A treatment of 10 *μ*mol/L of SAHA and 10 mmol/L of SB significantly decreased the relative live cell number to 52.8 ± 5.1% and 77.1 ± 5.0% compared to the control, respectively. In the presence of theophylline, the effects of SAHA and SB were significantly attenuated to 65.3 ± 4.8% and 86.8 ± 1.6%, respectively (Fig. 2B). These results suggest that the Hdac inhibitory activity of BPE is involved in the decrease in cell viability in Neuro2a cells. It raises two possibilities in the decrease in live cell number, induction of cell death, and/or inhibition of cell growth. To evaluate the possibility of cell death, BPE-treated cells were subjected to live/dead cell staining with acetoxy methylated-calcein and propidium iodide (PI) (for detailed methods, see Data S1). In untreated cells, the PI-positive dead cells were 0.2 ± 0.3%. While 200 *μ*g/mL of BPE treatment significantly increased PI-positive dead cells to 8.5 ± 4.3%. By addition of theophylline, the dead cells induced by BPE were significantly decreased to 3.4 ± 1.2% (Fig. 2C and D). These data indicate that BPE induced cell death in Neuro2a cells via Hdac inhibitory activity. To elucidate how BPE induced the cell death, we evaluated the effects of specific inhibitors against caspase-8 (Z-IETD-FMK) and caspase-9 (Z-LEHD-FMK) on BPE-induced cell death. The relative live cell number of 200 *μ*g/mL of BPE treatment was 40.3 ± 2.5% compared to the control. By addition of 10 *μ*mol/L of caspase-8 or -9 inhibitor, the effect of BPE was significantly attenuated to 54.8 ± 12.3% and 53.7 ± 5.2%, respectively (Fig. 2E). These results suggest that BPE induces apoptosis via both caspase-8 and -9 activations. BPE has been reported to induce apoptosis by increasing death receptor TRAIL-R2 expression (Szliszka et al. 2011). On the other hand, Hdac inhibitors have been reported to increase TRAIL-R1 and TRAIL-R2 expression (Fulda 2012). Caspase-8 is known to be involved in cell death signaling via death receptor, such as TRAIL-R (Crowder and El-Deiry 2012). Our data support previous finding that Brazilian green propolis induced TRAIL-dependent cell death and suggest that TRAIL-dependent cell death can be regulated by histone acetylation. In addition, caspase-9 is known to be involved in mitochondrial dysfunction-dependent cell death (Reubold and Eschenburg 2012). Hdac inhibitor has been reported to cause the loss of mitochondrial membrane potential that can cause cell death (Han et al. 2013). Therefore, our data suggest that BPE induce apoptotic cell death via both death receptor signaling and mitochondrial dysfunction by Hdac inhibition.

**Figure 2:**
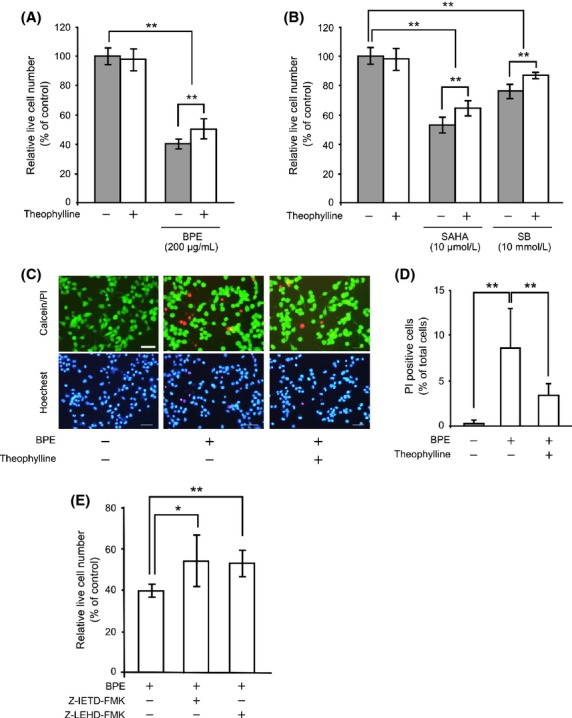
Effects of BPE on cell viabilities and its dependence on Hdac inhibitory activity. Neuro2a cells were treated with 200 *μ*g/mL of BPE (A), 10 *μ*mol/L of SAHA or 10 mmol/L of SB (B) in the presence or absence of 100 *μ*mol/L of theophylline for 24 h. Live cells were counted by the trypan blue exclusion assay. The results are presented as mean ± SD of relative live cell numbers compared to the control (*n* = 6). **P < 0.01, ANOVA. (C) Typical patterns of the live/dead cell staining subjected Neuro2a cells (Calcein/PI). Nuclei were stained with a Hoechst33342 (Hoechst). Scale bar: 50 *μ*m. (D) Relative ratios of PI-positive dead cells were calculated. The values were represented as the ratio of PI-positive dead cells in total cells ± SD (*n* = 12). **P < 0.01, ANOVA. (E) Neuro2a cells were treated with 200 *μ*g/mL of BPE in the presence or absence of 10 *μ*mol/L of caspase-8 inhibitor or caspase-9 inhibitor for 24 h. The results are presented as mean ± SD of relative live cell numbers compared to the control (*n* = 6).*P < 0.05, **P < 0.01, ANOVA. BPE, Brazilian propolis extract; Hdac, histone deacetylase; SAHA, suberoylanilide hydroxamic acid; SB, sodium butyrate; ANOVA, analysis of variance; PI, propidium iodide.

Regarding the effect on cell growth, we analyzed the effect of BPE treatment on cell cycle by imaging cytometric analysis. Neuro2a cells were treated with 200 *μ*g/mL of BPE for 24 h and then harvested. After that, the cells were fixed and stained with PI. The stained cells were subjected to cell cycle analysis using image-based cytometer (for detailed methods, see Data S1). The ratio of cell number at the Sub-G1, G1, S, and G2/M-phases were as follows: 1.7 ± 0.5%, 59.3 ± 3.1%, 18.0 ± 1.7%, and 20.0 ± 1.7% in untreated control cells; 9.0 ± 1.7%, 47.3 ± 2.8%, 13.0 ± 2.6%, and 30.3 ± 2.5% in BPE-treated cells; and 4.7 ± 1.1%, 51.7 ± 2.3%, 17.3 ± 4.9%, and 25.3 ± 1.5% in BPE and 100 *μ*mol/L theophylline-cotreated cells (Fig. 3), respectively. BPE treatment significantly increased the ratio at the Sub-G1 and G2/M-phase and decreased the ratio at G1-phase compared to the control. By addition of theophylline, the effect of BPE significantly attenuated (Fig. 3). These data suggest that BPE arrested cell cycle at the M-phase in Neuro2a cells via Hdac inhibition. In the previous reports, Hdac inhibitors such as Trichostatin A, SAHA, and SB have been shown to arrest the cell cycle at the M-phase in several neuroblastoma cell lines (Mühlethaler-Mottet et al. 2008; Francisco et al. 2012). It strongly supports our observation that Hdac inhibition by BPE arrested the cell cycle at the M-phase. Moreover, BPE treatment significantly increased the Sub-G1 ratio. Since the cells in Sub-G1 have reduced genomic DNA, these cells are thought as apoptotic cells. It supports the result that BPE treatment increased dead cells in live/dead cell staining (Fig. 2C and D). Our data indicate that BPE reduced cell viability by both inductions of cell death and cell cycle arrest via Hdac inhibition. Although BPE was previously reported to induce cell death and cell cycle arrest in various cancer cell lines (Kimoto et al. 1998; Li et al. 2007; Búfalo et al. 2009), our data are the first observation that Hdac inhibition by BPE contributes cell death and cell cycle arrest.

**Figure 3:**
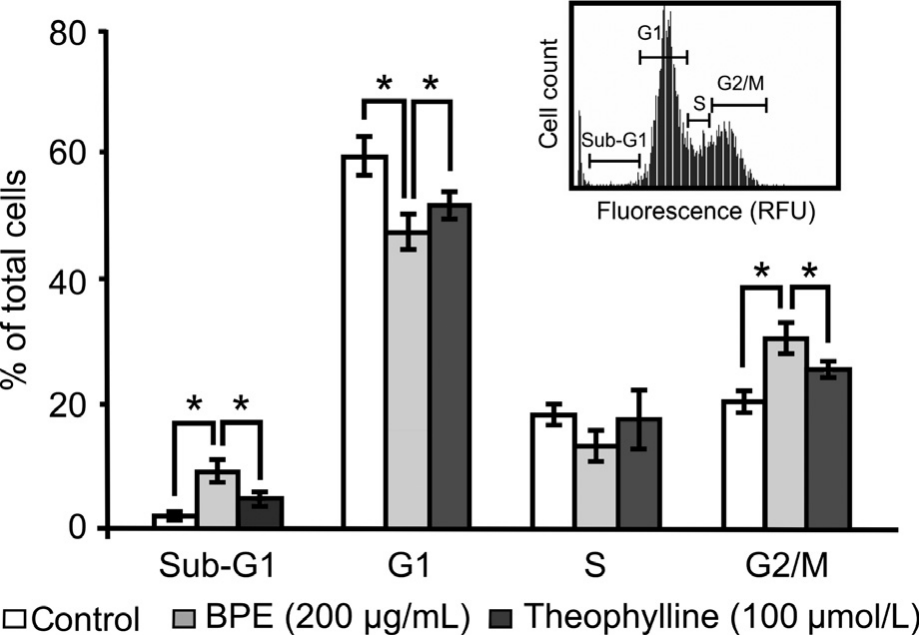
Effects of BPE on cell cycle and its dependence on Hdac inhibitory activity. Neuro2a cells were treated with 200 *μ*g/mL of BPE in the presence or absence of theophylline for 24 h. Inset shows typical pattern of imaging cytometric analysis of control cells. Percentages of the cell number in each phase were calculated. The values are represented as the mean of the ratio in each phase ± SD (*n* = 3). *P < 0.05, nonpaired *t*-test. BPE, Brazilian propolis extract; Hdac, histone deacetylase.

In conclusion, BPE inhibited class I Hdac enzyme activity and increased cellular histone acetylation in Neuro2a cells. BPE showed antitumor effects in Neuro2a cells by induction of cell death and cell cycle arrest at the M-phase via the Hdac inhibitory activity. This study is the first findings that Brazilian green propolis affects epigenetics in mammalian cells, and the epigenetic effect of BPE contributes to antitumor property.
