# Supplementary material for: Histone deacetylase inhibitory effect of Brazilian propolis and its association with the antitumor effect in Neuro2a cells
Source: Food Sci Nutr. 2014 Jun 2;2(5):565–70. doi: 10.1002/fsn3.131 (PMC4237486; doi:10.1002/fsn3.131)
Supplement: Supplementary file 1 — Data S1. Materials and Methods. [file fsn30002-0565-sd1.docx]

**Supporting Information**

**Material and methods**

**1. Materials**

Sodium butyrate was purchased from Johnson Matthey (London, UK). Theophylline was purchased from Nacalai tesque (Kyoto, Japan).

**2. Preparation of Brazilian propolis extract (BPE)**

10 g of raw Brazilian propolis (Minas Gerais state, Brazil) was cut into small pieces and then extracted with 50 ml of 80% ethanol at room temperature for three days. The suspension was centrifuged at 3000 rpm for 10 min. The supernatant was concentrated under reduced pressure and was lyophilized to give 3.73 g of powder. It was dissolved in dimethyl sulfoxide (DMSO) and stored at -5°C.

**3. Cell culture and treatment**

Mouse neuroblastoma Neuro2a cells were obtained from the Health Science Research Resources Bank (Osaka, Japan), and maintained in the Dulbecco’s modified Eagles medium (DMEM) (Sigma, St. Louis, MO, USA) supplemented with 10% heat-inactivated fetal bovine serum (FBS), 100 units/ml penicillin, and 100 μg/ml streptomycin (Sigma) at 37°C in a humidified 5% CO_2_ atmosphere. Regarding cell treatment, Neuro2a cells were cultured at 37°C in a humidified 5% CO_2_ atmosphere for 24 h. After removal of DMEM, the cells were treated with a variable concentration of BPE in fresh DMEM for 6 or 24 h. Then, the cells were subjected to various analyses.

**4. Hdac enzyme activity assay**

Hdac enzyme activity was analyzed by CycLex HDACs fluorometric assay kit (CycLex, Nagano, Japan) under manufacturer's instruction. Briefly, a variable concentration of 10 μl of BPE or solvent blank was applied into each well of a 96-well plate. Then 10 μl of a crude HDACs solution and 30 μl of an assay buffer (aqueous solution of 0.25 mAU/ml lysylendopeptidase and 20 μM fluoro-substrate peptide) were added. The plate was incubated for 30 min at 37°C. Then 5 μM of trichostatin A was add to each well to terminate the reaction. The excitation wavelength at 355 nm and the emission wavelength at 460 nm were measured by a fluorescent microplate reader (Molecular Devices, Silicon Valley, CA, USA).

**5. Histone acetylation assay**

The cellular histone acetylation was quantitated by a Cellular histone acetylation assay kit (CycLex) under the manufacturer's instruction. Briefly, Neuro2a cells were cultured in a 96-well plate at a cell density of 5,000 cells/well. After 24 h, the cells were treated with 100 or 200 μg/ml of BPE for 6 h, and then washed with PBS. After that, the cells were fixed with ice-cold 95% (v/v) methanol, blocked and incubated with a primary antibody solution (anti-acetylated histone/p53-K382 monoclonal antibody TM-5C5). After washing, the cells were incubated with a secondary antibody solution (HRP conjugated anti-mouse IgG), followed by incubation using a substrate reagent. The incubation was terminated by addition of stop solution. The absorbance at 450 nm was measured by a microplate reader (Molecular Devices). To calculate the acetylation per live cell number, relative acetylation was corrected by the cell viability. The cell viabilities were measured by a 3-(4, 5-dimethyl-2-thiazolyl) 2, 5-diphenyl-2-H-tetrazolium bromide (MTT) assay under same condition. Briefly, Neuro2a cells were cultured and treated with BPE. Then, 0.25 mg/ml of MTT in serum free DMEM were added onto the cells and incubated for 2 h. The incubation was terminated by addition of 20% (w/v) sodium dodecylsulfate and 50% (v/v) dimethylformamide in water. The absorbance at 570 nm was measured by a microplate reader.

**6. Western blotting**

Neuro2a cells were harvested and lysed in PBS containing a 1% protease inhibitor cocktail, and followed by homogenization with a Dounce homogenizer. The cell suspensions were centrifuged at 1,000 × g for 10 min at 4°C. The precipitate, nuclei fraction was lysed in PBS containing a 1% protease inhibitor cocktail and 1% TritonX-100, and protein concentration was assayed by a protein quantification kit (Dojindo, Kumamoto, Japan). The samples of the control and the treated cells were separated by 15% SDS-polyacrylamide gel electrophoresis, followed by transfer to a polyvinylidene fluoride (PVDF) membrane. The membranes were blocked with 3% skimmed milk and 0.1% Tween20 in phosphate-buffered saline (PBS) and incubated at 4°C overnight with anti-histone H3 (dilution, 1:1000) and anti-acetyl (Ac) histone H3 (Lys9 / Lys14) (dilution, 1:1000) (Cell signaling technology, Beverly, MA, USA). After washing, the membranes were incubated with a biotin-conjugated secondary antibody (dilution, 1:1000) (Nacalai tesque), followed by incubation with a streptavidin biotin complex peroxidase kit (Nacalai tesque). The immunoreactivity was visualized using a peroxidase detection kit (Nacalai tesque).

**7. Live/dead cell staining**

Live or dead cell staining was performed by Calcein-AM and PI (Dojindo) double staining. Neuro2a cells were cultured in a poly-lysine coated 24-well plate at a cell density of 30,000 cells/well. After 24 h of treatment with BPE, the cells were stained with 0.5 μg/ml of Calcein-AM and 0.5 μg/ml of PI at 37°C for 30 min. Nuclei were stained with 0.5 μg/ml of Hoechest33342 (Dojindo). Stained cells were observed under fluorescent microscopy. PI positive cells were considered as dead cells.

**8. Cell cycle analysis**

BPE treated Neuro2a cells were harvested and washed twice with a phosphate-buffered saline (PBS). Then, the cells were fixed with a 70% (v/v) ice-cold ethanol at 4°C overnight. After washing, the fixed cells were digested with 0.2 mg/ml of RNase A in PBS at room temperature overnight. The cells were stained with 20 μg/ml of PI at 37°C for 30 min. The stained cells were subjected to cell cycle analysis using a Tali image-based cytometer (Life technologies, Carlsbad, CA, USA).

**9. Statistical analysis**

The data are represented as mean ± S.D. The differences were analyzed using one -way ANOVA followed by Tukey-Kramer post-hoc test or non-paired *t*-test. The *p*-values less than 0.05 were considered to be significant.
